# Optimal Clinical Target Volume of Radiotherapy Based on Microscopic Extension around the Primary Gross Tumor in Non-Small-Cell Lung Cancer: A Systematic Review

**DOI:** 10.3390/cancers14092318

**Published:** 2022-05-07

**Authors:** Yukihisa Tamaki, Norihiro Aibe, Takafumi Komiyama, Satoshi Nagasaka, Toshiyuki Imagumbai, Tomoko Itazawa, Hiroshi Onishi, Tetsuo Akimoto, Yasushi Nagata, Yuko Nakayama

**Affiliations:** 1 Department of Radiation Oncology, Shimane University Faculty of Medicine, 89-1 Enya-cho, Izumo-shi 693-8501, Shimane, Japan; 2 Japanese Radiation Oncology Study Group, 2-17-8, Higasi Komagata, Sumida-ku 130-0005, Tokyo, Japan; takafumi@yamanashi.ac.jp (T.K.); honishi@yamanashi.ac.jp (H.O.); takimoto@east.ncc.go.jp (T.A.); nagat@hiroshima-u.ac.jp (Y.N.); yukonak4@ncc.go.jp (Y.N.); 3 Department of Radiology, Kyoto Prefectural University of Medicine, 465 Kajii-cho, Kamigyo-ku, Kyoto-shi 602-8566, Kyoto, Japan; a-ib-n24@koto.kpu-m.ac.jp; 4 Department of Radiology, University of Yamanashi Faculty of Medicine, 1110 Shimokato, Chuo-shi 409-3898, Yamanashi, Japan; 5 Department of General Thoracic Surgery, National Center for Global Health and Medicine, 1-21-1 Toyama, Shinjuku-ku 162-8655, Tokyo, Japan; snagasak@hosp.ncgm.go.jp; 6 Department of Radiation Oncology, Kobe City Medical Center General Hospital, 2-1-1 Minatojima Minamimachi, Chuo-ku, Kobe-shi 650-0047, Hyogo, Japan; gumbai@kcho.jp; 7 Department of Radiation Oncology, St. Luke’s International Hospital, 9-1 Akashi-cho, Chuo-ku 104-8560, Tokyo, Japan; itatomok@luke.ac.jp; 8 Division of Radiation Oncology and Particle Therapy, National Cancer Center Hospital East, 6-5-1 Kashiwanoha, Kashiwa-shi 277-8577, Chiba, Japan; 9 Department of Radiation Oncology, Graduate School of Biomedical Sciences, Hiroshima University, 1-2-3 Kasumi, Minami-ku, Hiroshima-shi 734-8553, Hiroshima, Japan; 10 Department of Radiation Oncology, National Cancer Center Hospital, 5-1-1 Tsukiji, Chuo-ku 104-0045, Tokyo, Japan

**Keywords:** non-small-cell lung cancer, radiation therapy, gross tumor volume, systematic review

## Abstract

**Simple Summary:**

As radical radiation therapy for non-small-cell lung cancer becomes increasingly precise following the development of techniques such as image-guided radiotherapy and intensity-modulated radiotherapy, how we define the clinical target volume (CTV) is crucial. Although assessing the scope of microscopic extension and microscopic proximal bronchial extension from the primary tumor is critical for defining the CTV, there is currently no consensus on this issue. Accordingly, we conducted this systematic review with the aim of assessing the available evidence. Although there were few studies with a high level of evidence, this systematic review enabled us to collate the available results and to provide some recommendations regarding suitable CTV margins.

**Abstract:**

A crucial issue in radical radiation therapy for non-small-cell lung cancer is how to define the clinical target volume (CTV). Although the scope of microscopic extension (ME) and microscopic proximal bronchial extension (PBE) from a primary tumor should be considered when defining the CTV, there has been limited research on ME and PBE. Therefore, we conducted this systematic review. The PubMed, ICHUSHI (Japanese database), and Cochrane Library databases were searched, and 816 articles were initially retrieved. After primary and secondary screenings, eight articles were ultimately selected. The results of this systematic review suggest the importance of a 0 mm margin in stereotactic radiotherapy for early-stage cancer and a 5–8 mm margin in curative irradiation for locally advanced cancer. Regarding PBE, this review yielded the conclusion that it is appropriate to consider the addition of an approximately 15 mm margin from the bronchial vasculature. Although there were few articles with a high level of evidence, this systematic review enabled us to collate results from previous studies and to provide recommendations, to some extent, regarding the CTV margin in the current clinical environment, where high-precision radiation therapy, such as image-guided radiotherapy and intensity-modulated radiotherapy, is predominant.

## 1. Introduction

In multimodality therapy for non-small-cell lung cancer (NSCLC), radiotherapy is considered the standard therapy for patients with locally advanced or early-stage, but inoperable, disease [1]. High-precision radiotherapy has become widespread, with the establishment of radiation treatment planning based on computed tomography (CT) imaging, the introduction of high-precision algorithms, and the development of high-performance radiotherapy devices. However, the definition of an appropriate clinical target volume (CTV) that accounts for microscopic extension (ME) remains challenging.

In NSCLC, ME occurs around the gross macroscopic primary tumor [2]. Although the scope of ME from a primary tumor should be considered when defining the CTV, there has been limited research conducted on ME and few review articles have been published. Consequently, it is currently difficult to set an appropriate CTV. Accordingly, a review of the current literature on ME from the primary tumor is needed to provide useful information for defining the CTV for the primary tumor. Here, we conducted a systematic review of ME from the primary tumor in NSCLC and summarized the findings.

## 2. Materials and Methods

**Figure 1:**
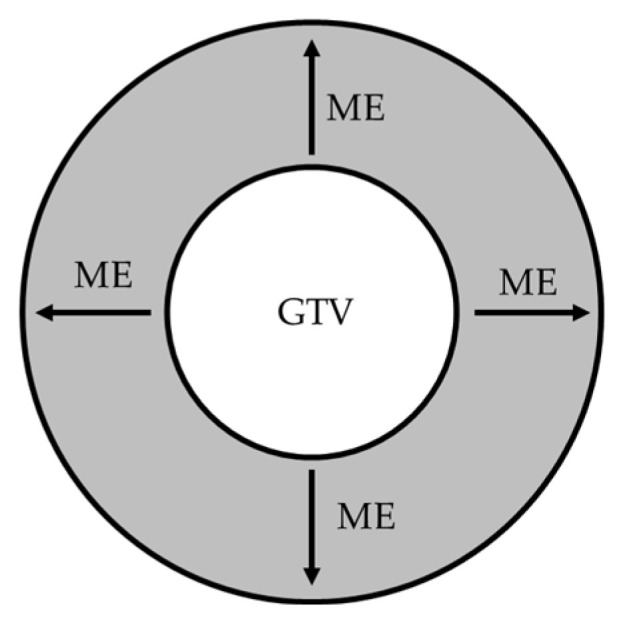
A schematic figure illustrating the GTV-to-CTV margin for ME into the lung parenchyma. GTV, gross tumor volume; CTV, clinical target volume; ME, microscopic extension.

**Figure 2:**
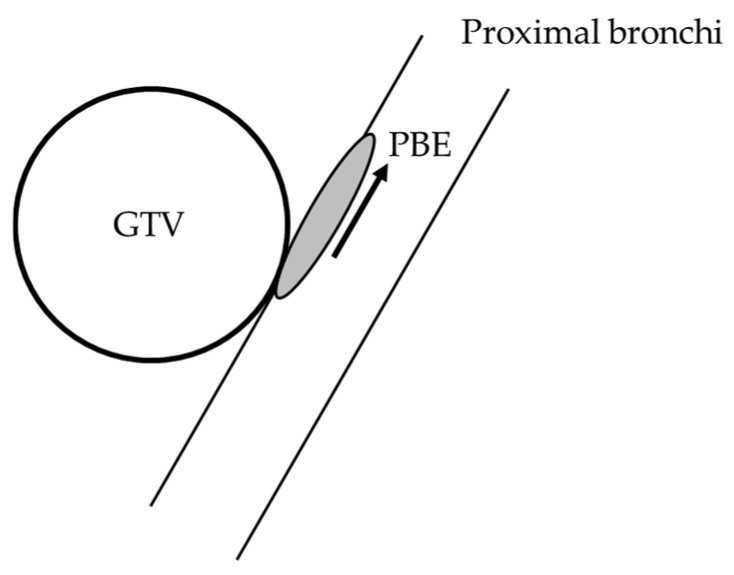
A schematic figure illustrating the GTV-to-CTV margin for microscopic PBE. GTV, gross tumor volume; CTV, clinical target volume; PBE, proximal bronchial extension.

The study was registered with the Research Registry and its unique identifying number (UIN) is: review registry 1355. We searched the PubMed, ICHUSHI (Japanese database), and Cochrane Library databases for articles published between 1997 and 2016. The search terms are shown in Supplemental data 1. Primary screening was performed based on the titles and abstracts of the retrieved articles in reference to the search terms. In addition to the articles found using these search terms, two experts performed manual searches to select other relevant articles for secondary screening. In the secondary screening, the full text was reviewed by the two experts to select articles for inclusion in the systematic review. Articles selected by only one expert were re-evaluated until a consensus was reached. Reports judged as relevant by both experts were included in the final selection. The selected articles were broadly classified into two categories: (1) ME from the gross primary tumor into the lung parenchyma, and (2) microscopic proximal bronchial extension (PBE) from the gross primary tumor. A schematic figure illustrating the GTV-to-CTV margin for ME into the lung parenchyma is shown in Figure 1, and for microscopic PBE in Figure 2, respectively. The results were extracted and summarized for review. This work was reviewed and approved by the Pulmonary and Mediastinal Tumors Committee of the Japanese Radiation Oncology Study Group (JROSG). The status of the work was also reported periodically at JROSG meetings.

## 3. Results

### 3.1. Search Results

**Figure 3:**
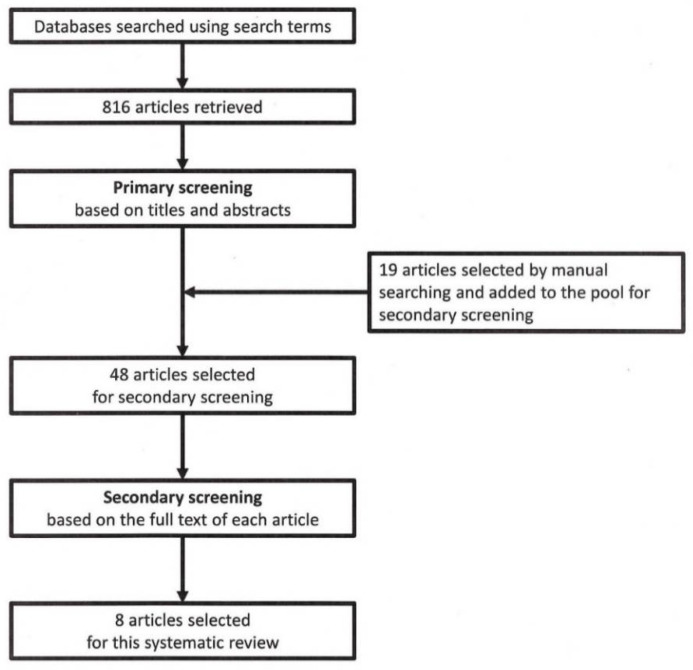
Flowchart of this systematic review.

A total of 816 articles were retrieved using the search terms. In the primary screening, 48 articles were chosen for further screening, comprising the following: 29 were selected based on their titles and abstracts, and an additional 19 were selected by manual searching. From the secondary screening, eight relevant articles with pathologic findings on ME from the primary tumor in NSCLC were ultimately selected [2,3,4,5,6,7,8,9]. A flowchart of the review process is shown in Figure 3. The eight articles could be broadly classified into two categories: (1) ME from the gross primary tumor into the lung parenchyma, and (2) microscopic PBE from the gross primary tumor. The results from these two categories were summarized separately, as shown in Table 1 and Table 2, respectively.

### 3.2. Microscopic Extension from the Gross Primary Tumor into the Lung Parenchyma

Giraud et al. [2] reported the analysis results from 70 patients (38 with squamous cell carcinoma and 32 with adenocarcinoma). They sorted the obtained slides and selected suitably evaluable slides from 42 patients (99 slides from 19 patients with squamous cell carcinoma and 123 slides from 23 patients with adenocarcinoma). They measured ME from the gross tumor volume. The mean ME differed significantly between two histologic types: 1.48 mm for squamous cell carcinoma (standard deviation [SD]: 2.37 mm) and 2.69 mm (SD: 2.76 mm) for adenocarcinoma. The maximum ME was 13 mm. Tumor coverage with a 5 mm margin from the pathological GTV was 91% for squamous cell carcinoma and 80% for adenocarcinoma. They concluded that the optimal margin from the pathological GTV to achieve 95% coverage of ME would be 6 mm for squamous cell carcinoma and 8 mm for adenocarcinoma. They also evaluated the relationship between the GTV on CT and the pathological GTV, but this was limited to a correlation analysis and did not describe how to define the GTV margins on CT.

Goldstein et al. [3] analyzed 31 patients with early lung adenocarcinoma (T1N0M0), and found that the mean ME distance was 7.4 mm (SD: 2.9 mm) and the median ME was 7 mm (range: 3–14 mm). They also found that ME tended to be greater in tumors of a lower histological grade. Grills et al. [4] investigated 36 patients with early lung adenocarcinoma (T1N0M0), and found that the mean ME distance was 7.2 mm (SD: 3.1 mm; range: 2–16 mm). Similarly to Goldstein et al. [3], they also found that lower-grade tumors were more likely to have greater ME, which was attributed to lepidic growth. They also found that the radiologic GTV contoured manually using CT lung windows tended to be larger than the pathological GTV, although the radiologic GTV underestimated the CTV (pathological GTV + ME) by 1.2 ± 9 mm. They concluded that it would be necessary to add a 9 mm margin to the radiologic GTV on CT lung windows, in order to cover the pathological CTV (pathological GTV + ME) for all grades (90% confidence). However, the required margin differed significantly according to the histological grade, as follows: grade 1: 9 mm; grade 2: 7 mm; and grade 3: 4 mm (80% confidence, due to the small sample size).

Stroom et al. [5] analyzed five patients with lung cancer (stage I–III; three patients with squamous cell carcinoma, one with adenosquamous carcinoma, and one with sarcomatoid carcinoma), and found that the mean ME was 5 mm when measured without the correction of deformations that occur during specimen fixation, and 9 mm with correction of the deformations (median: 5 mm; range: 0–9 mm). The GTV delineated on CT was larger than the pathological GTV in three out of five patients, while the CT-based GTV and the pathological GTV were comparable in one patient. Thus, the GTV delineated on CT was either comparable with or greater than the pathological GTV in 80% of patients. In addition, they reported that the GTV delineated on positron emission tomography (PET) (SUV threshold: 42%) showed a much better correlation with the pathological GTV than the GTV delineated on CT.

Van Loon et al. [6] analyzed 34 patients with NSCLC (18 patients with adenocarcinoma, 6 with squamous cell carcinoma, 4 with large cell carcinoma, 3 with mixed-type adenocarcinoma, 1 with bronchioloalveolar carcinoma, and 2 with other types of NSCLC). They compared GTV and CTV determined pathologically and via imaging (CT and PET). They observed ME in 50% of patients (17/34) and concluded that the CTV margin necessary to cover ME in 90% of patients was 14 mm without deformation correction and 26 mm with deformation correction. In addition, their multivariate analysis showed that the volume and mean CT value of the gross tumor on CT were factors that indicated possible ME. Meng et al. [7] evaluated the relationship between ME and PET/CT imaging findings in 39 patients with NSCLC (stage I–III; 22 patients with adenocarcinoma and 17 with squamous cell carcinoma; 13 patients with grade 3, 16 with grade 2, and 10 with grade 1). They analyzed the correlations among ME, maximal standardized uptake values (SUVmax), metabolic tumor volume (MTV), and other clinical pathologic parameters. The mean ME was 3.38 mm (SD: 2.80 mm; range: 0–13 mm), with no significant difference between squamous cell carcinoma and adenocarcinoma. However, lower-grade tumors showed less extensive ME. Positive correlations were found between ME and SUVmax, ME and MTV (defined as the tumor volume with SUV over a threshold of 2.5), and SUVmax and MTV. Therefore, the authors concluded that the margin that would need to be added to the GTV for the CTV to cover ME in 95% of patients would be 1.93 mm at SUVmax ≤ 5, 3.90 mm at SUVmax 5–10, and 9.6 mm at SUVmax > 10, as well as 1.92 mm at MTV ≤ 10 cm^3^, 3.80 mm at MTV 10–70 cm^3^, and 10.90 mm at MTV > 70 cm^3^.

### 3.3. Microscopic Proximal Bronchial Extension from the Gross Primary Tumor

Kara et al. [8] evaluated PBE by pathological examination in surgical specimens from 70 patients with NSCLC (38 patients with squamous cell carcinoma, 15 with adenocarcinoma, 8 with bronchioloalveolar carcinoma, 6 with adenosquamous cell carcinoma, and 3 with large-cell carcinoma). Microscopic PBE was observed in 24.2% of the patients (17/70), and the range of PBE was 0 to 3 mm. They concluded that a 10 mm margin would have covered PBE in 86% of cases, and a 15 mm margin would have covered PBE in 93% of cases. They also found that the frequency of PBE was higher in squamous cell carcinoma than adenocarcinoma, but the PBE distance was longer in adenocarcinoma. In a separate article, Kara et al. also analyzed the differences in PBE by tumor location [9]. Overall, the mean PBE was 10.94 mm (SD: 7.07 mm). PBE was more frequent in central tumors than peripheral tumors (30.3% vs. 18.9%), but the PBE distance was longer in peripheral tumors (mean ± SD = 7.60 mm ± 3.47 mm vs. 15.71 mm ± 8.38 mm). Therefore, the authors noted that although the typical bronchial safety margin of about 20 mm used in surgical resection is practical, some peripheral tumors may extend beyond 20 mm.

## 4. Discussion

Lung cancer is one of the primary causes of cancer mortality worldwide [10,11], and the course of treatment is determined according to the tumor stage, nodal stage, and extrapulmonary metastases [12,13]. Curative radiotherapy is an important treatment option for inoperable localized lung cancer, and appropriate definitions of the GTV and CTV are extremely important when determining the size of the radiation field for radiotherapy.

For this review, we searched for English- and Japanese-language articles published between 1997 and 2016, and found no studies on ME from GTV with a high level of evidence (e.g., systematic reviews, randomized controlled trials, or meta-analyses). We also found that the methods used to evaluate ME in comparison with CT imaging were not well established, and the methods used to measure ME differed between researchers and institutions. Therefore, we cannot definitively state the optimal GTV-CTV margin for primary tumors of NSCLC. However, we can draw some conclusions based on a summary of this systematic review. Although tentative, these conclusions are valuable because these studies were published sporadically, and their results have not been summarized previously.

### 4.1. ME from the Gross Primary Tumor into the Lung Parenchyma

The studies we reviewed show that ME beyond the pathological GTV can range from 0 to 16 mm, except for the report by van Loon et al. The mean values of ME reported in these studies indicate that a 5 mm margin would cover tumor extension in over 50% of patients, and Giraud et al. suggest that a 5 mm margin would cover tumor extension in over 80% of patients [2]. Based on the reports by Giraud, Grills, and Meng, the clinical target volume could be expanded by about 9 mm from the GTV, in order to cover ME in 90–95% of cases [2,4,7]. However, van Loon et al. [6] found that a larger extension (26 mm) was needed to achieve such coverage. This difference may be attributable to methodological differences in the evaluation of ME, but the results from their study [6] suggest that some tumors may extend microscopically over a long distance of up to 20–30 mm. According to the European Society for Radiotherapy and Oncology (ESTRO) Advisory Committee in Radiation Oncology guidelines, a suitable CTV margin for locally advanced NSCLC is recommended to be 5–8 mm [14].

On the other hand, the values of SUVmax on PET imaging might be useful for predicting the extent of ME. Meng et al. suggested the potential of using SUVmax values to predict ME [7].

Consequently, considering the radiation doses to organs at risk, the addition of a 5 mm to 9 mm margin to the GTV on imaging seems to be appropriate for delineating the CTV. These margins could also be modified based on SUVmax. However, as noted by Grills et al. [4] and Giraud et al. [2], the potential to achieve the control of microscopic disease with a lower radiation dose than that prescribed for the GTV should also be considered. Furthermore, Grills et al. reported that dosimetric coverage of 6 mm to 8.5 mm ME could be achieved by adding a margin of 3 mm to 5 mm to the GTV for a prescribed dose of 48 Gy, divided into four fractions [4]. In addition, Jin et al. reported that the potential CTV may incidentally receive adequate and relatively homogeneous doses when the internal target volume is used and patients have large respiratory motion. However, this could result in underdosing for gated treatment or patients with small respiratory motion [15]. Thus, when defining the CTV while taking ME into account, close attention should be paid to avoid unduly expanding the CTV margins. Indeed, clinical trials of stereotactic body radiation therapy for early NSCLC have demonstrated excellent durable local tumor control of over 90%, with no margin added to the GTV in setting the CTV in the NRG Oncology Radiation Therapy Oncology Group studies [16,17]. The results of good disease control in these trials with no GTV-CTV margin suggest that a lower dose on the penumbra of the dose distribution might unintentionally cover and control microscopic disease.

In summary, the results of this review show that a GTV with a margin of 5–9 mm covers a reasonable range of ME. However, the optimal prescribed dose for ME lesions has not yet been clarified, including whether the GTV and the ME lesions should receive the same dose. For this reason, at present, it is important perform treatment with a 0 mm margin for stereotactic radiotherapy in early-stage cancer and a 5 mm to 8 mm margin for curative irradiation for locally advanced NSCLC, in line with the treatment protocol of previous clinical trials [16,17] and the ESTRO guidelines [14]. While continued research is obviously needed, the results of this review at least suggest that a margin of 5–9 mm from the GTV may provide greater local control.

### 4.2. Microscopic Proximal Bronchial Extension from the Gross Primary Tumor

In this systematic review, the only studies of PBE were those by Kara et al. [8,9], and, thus, we were unable to validate the results. However, according to their reports, PBE was observed in approximately one out of four patients, and a margin of about 15 mm was found to cover PBE in over 90% of patients. Therefore, it should be appropriate to consider the addition of an approximately 15 mm margin from the bronchial vasculature to cover possible PBE, while also considering the radiation doses to organs at risk.

## 5. Conclusions

Following our objective systematic review of the literature, we have presented the results on MEs reported in the identified studies, as well as providing our own recommendations for CTV margins based on those results. The main limitations of our study were that our search yielded few relevant articles, and that none of the articles had a high level of evidence. However, this was unavoidable, due to the small body of research available on the topic.

In light of this lack of high-level evidence, we believe that conducting this systematic review contributes to the literature by organizing the findings of previous studies and providing recommendations, to some extent. Now that new modalities targeting tumors more precisely are increasingly being used as curative radiotherapy for lung cancer, such as image-guided radiotherapy and intensity-modulated radiotherapy, an accurate definition of CTV margins and knowledge of the optimal dose for controlling microscopic disease around the gross tumor will likely become even more important. Additional findings from randomized trials and other such research with high levels of evidence will be necessary for further investigation of this topic.

## Figures and Tables

**Table 1:** Studies reporting ME into the lung parenchyma.

Author (Year Published)	Number of Patients	Patient Characteristics	Analysis Results for ME Measured from Pathological GTV
Giraud (2000) [2]	42 *	AC 23/SCC 19 Stage 1–4	Mean (SD): AC 2.69 mm (2.76 mm), SCC 1.48 mm (2.37 mm) Range: AC 0–12 mm, SCC 0–13 mm 5-mm margin coverage: AC 80%, SCC 91% 95% coverage margin: AC 8 mm, SCC 6 mm
Goldstein (2003) [3]	31	AC 31 Stage 1 (T1N0M0) G1/G2/G3 = 12/12/7	Mean (SD): 7.4 mm (2.9 mm) Median: 7 mm (range: 3.0–14.0 mm)
Grills (2007) [4]	36	AC 36 Stage 1 (T1N0M0) G1/G2/G3 = 11/15/10	Mean (SD): 7.2 mm (3.1 mm) Range: 2–16 mm Pathological GTV-CTV margin (90% of cases): all grades, 12.5 mm Pathological GTV-CTV margin: G1 13 mm, G2 9.7 mm, G3 4.4 mm Radiologic GTV-CTV margin (90% confidence): all grades, 9 mm Radiologic GTV-CTV margin (80% confidence): G1 9 mm, G2 7 mm, G3 4 mm
Stroom (2007) [5]	5	SCC 3/SCC + AC 1/sacromatoid + AC 1 Stage 1–3	Median: 5 mm (range, 0–9 mm) Mean: 5 mm (without deformation correction) Mean: 9 mm (with deformation correction)
van Loon (2012) [6]	34	AC 18/SCC 6/LCC 4/Others 6	ME observed in 50% of patients (17/34) 90% coverage margin: 14 mm (without deformation correction) 90% coverage margin: 26 mm (with deformation correction)
Meng (2012) [7]	39	SCC 17/AC 22 Stage 1–3 G1/G2/G3 = 10/16/13	Mean (SD): 3.38 mm (2.80 mm) Range: 0–13 mm 95% coverage margin: 1.93mm (SUVmax ≤ 5), 3.90 mm (SUVmax 5–10), 9.60 mm (SUVmax > 10)

ME, microscopic extension; GTV, gross tumor volume; CTV, clinical target volume; AC, adenocarcinoma; SCC, squamous cell carcinoma; GX, grade X; LCC, large-cell carcinoma; SD, standard deviation; SUVmax, maximal standard uptake value. * The total number of patients was 70, but the results reported were those for the 42 analyzed patients with easily evaluable slides.

**Table 2:** Studies reporting microscopic PBE.

Author (Year Published)	Number of Patients	Patient Characteristics	Results from Analysis of PBE
Kara (2000) [8]	70	SCC 38/AC 23 ASC 6/LCC 3 Central tumors: 33 Peripheral tumors: 37	PBE observed in 17/70 patients (24.2%) PBE positive rate: central 30.3%, peripheral 18.9% Mean (SD) [overall]: 10.94 mm (7.07 mm) Mean (SD) [central]: 7.60 mm (3.47 mm) Mean (SD) [peripheral]: 15.71 mm (8.38 mm)
Kara (2001) [9]	70	SCC 38/AC 15 /BAC 8/ASC 6/LCC 3	PBE observed in 17/70 patients (24.2%) Range: 0–3 cm 1.0 cm margin coverage: 86% 1.5 cm margin coverage: 93%

PBE, proximal bronchial extension; AC, adenocarcinoma; SCC, squamous cell carcinoma; ASC, adenosquamous carcinoma; LCC, large-cell carcinoma; BAC, bronchioloalveolar carcinoma; SD, standard deviation.

## Supplementary Materials

The following are available online at https://www.mdpi.com/article/10.3390/cancers14092318/s1, Supplemental data 1: The search terms.
