# Supplementary material for: Optimal Clinical Target Volume of Radiotherapy Based on Microscopic Extension around the Primary Gross Tumor in Non-Small-Cell Lung Cancer: A Systematic Review
Source: Cancers (Basel). 2022 May 7;14(9):2318. doi: 10.3390/cancers14092318 (PMC9103011; doi:10.3390/cancers14092318)
Supplement: Supplementary file 1 [file cancers-14-02318-s001.zip › cancers-1710817-supplementary.pdf]

Supplemental data 1.

We searched the PubMed, ICHUSHI (Japanese database), and Cochrane Library da-tabases for articles published between 1997 and 2016. The search was performed on September 11, 2017.

The search terms are as follows.

1) PubMed database
("Lung Neoplasms/radiotherapy"[Mesh] OR ("Lung Neoplasms"[Mesh] AND "Radiotherapy"[Mesh]) OR "Lung Neoplasms/pathology"[Mesh] OR ((Lung Neoplasm*[TIAB] OR Pulmonary Neoplasm*[TIAB] OR Lung Cancer*[TIAB] OR Pulmonary Cancer*[TIAB] OR Lung Carcinoma*[TIAB]) AND (Radiotherap*[TIAB] OR Radiation Therap*[TIAB] OR Irradiation*[TIAB] OR radiation*[ALL]))) AND (target volume*[TIAB] OR CTV[TIAB]) AND 1997:2016[DP] AND (JAPANESE[LA] OR ENGLISH[LA])

2) ICHUSHI database (Japanese database)
("Hai-Syuyou;Housyasen-Ryouhou"/TH or ("Hai-Syuyou;Tiryou"/TH and Housyasen-Ryouhou/TH) or ((Hai-Syuyou/TA or Hai-Gan/TA or Hai-Gan/TA or Hai-Gan/TA or Hai-Akuseisyuyou/TA) and (Housyasen/TA or Syousya/TA or Densisen/TA))) and (Rinsyouteki-Hyouteki-Taiseki/TA or Rinsyouteki-Hyouteki-Taiseki/TA or (clinical/TA and target/TA and (Taiseki/TH or volume/TA)) or CTV/TA) and (DT=1997:2016)

3) Cochrane Library database (CDSR, CCRCT)
(Lung Neoplasm or Pulmonary Neoplasm or Lung Cancer or Pulmonary Cancer or Lung Carcinoma) and (Radiotherapy or Radiation Therapy or Irradiation or radiation) and (target volume or CTV): Publication Year from 1997 to 2016
